# Stomach and Intestines

**Published:** 1905-11-18

**Authors:** 


### PROGRESS IN MEDICINE AND SURGERY.

### STOMACH AND INTESTINES.

(*Continued from page 103.*)

**The Physiology of Nutrition.**—At a recent meeting of the American Gastro-enterological Association, Mendel[3] drew attention to the complexity of the digestive processes demonstrated by modern biochemistry, and emphasised the important place of ferments in all the newer conceptions of physiological processes. Ferments or enzymes are special types of catalytic agents, colloidal in nature, resembling the inorganic catalyzers in their action, and possessing the power of reverse action under altered environment. Allchin's Lumleian Lectures[4] are an elaborate and exhaustive exposition of the present position of physiological chemistry as far as it deals with nutrition and its perversions. His lectures are far too lengthy for abstraction or even summary in these columns, but his main points may be briefly stated, as they present a valuable picture of the views of recent investigators. Allchin holds that the primary foodstuffs, carbohydrate, fat and proteid, first undergo, as the result of intestinal ferment action, a process of hydrolysis by which without much loss of potential energy they become fit for absorption. In their passage through the intestinal wall this process is continued, especially by the proteids, though in the case of the fats some synthesis begins here. In the liver further synthesis of fats and carbohydrates, and possibly of proteids, takes place. Before utilization by the tissues the foodstuffs are in varying degrees stored up in insoluble form in different organs. Their conversion into soluble products capable of assimilation by the tissue cells is also the result of ferment action. Allchin emphasised the reversible action of ferments, the power namely which they possess of acting as katabolic or anabolic agents according to slight differences in their surroundings, such as change in osmotic pressure and the presence of excess of their own products. He also laid stress on the importance of the intracellular ferments by which the body cells are built up, each cell having the power of constructing its own particular form of protoplasm from the same kind of pabulum. Going a step further, he said that ferment action possibly depended on ionic action, simple chemical ions being the carriers of electric energy, and thus forming a link between inorganic chemical processes and the complex "vital" changes which characterise living matter. Going on to consider the subject of malnutrition, Allchin pointed out that when due to deprivation of food this was usually a matter of proteid or fat starvation, and that practically deficiency of carbohydrate in diet was seldom met with. This, no doubt, depends on economic conditions. Allchin does not consider that Chittenden's recent experiments, tending to show that average dietaries are much higher than the needs of the individual under healthy conditions, can be absolutely accepted and acted upon, and in this connection he has the support of many English physiologists and clinicians. He also points out that body-weight alone is not a sufficient criterion of nutrition; great individual variations occur, and the general condition of the tissues and the vigour of the mental and bodily powers must be taken into consideration. With regard to nutritional disturbances due to overfeeding, he shows that whereas fats in excess are largely excreted as such in the fæces, carbohydrates are absorbed and stored as fat and glycogen, sugar occasionally reappearing, as in alimentary glycosuria, in the urine. With proteids, too, absorption of the excess takes place, with increased elimination of urea and the products of cleavage known as purin bodies. It is when we come to disorders of nutrition, subsequent to assimilation, that the real difficulties begin, for the least derangement of one of the complex processes which intervene between the absorption of the food stuff from the intestine, and its ultimate incorporation in the cell, may set up a long chain of disturbances reacting all through the body. These disturbances appear to fall into groups corresponding to the various kinds of food-stuffs, and mainly recognised by variation in the excreta (*e.g.* glycosuria, increased uric acid excretion). Poisonous substances such as the toxins produced by fevers, or the dextrose present in diabetic blood, thus affect general nutrition by interfering with ferment actions in the tissues, while imperfect oxygen supply acts probably in two ways, firstly by allowing the formation of poisonous intermediate products, and secondly by preventing the occurrence of the normal oxidising actions by which substances of higher potential energy are formed for the use of the body. Finally Allchin deals in a most suggestive and interesting manner with the subject of atrophy and death. As life and nutrition seem dependent on the delicate action of innumerable enzymes, and as these enzymes are capable of reversed action under slight differences of environment, it is not difficult to suppose that greater inversions of normal ferment action bring about atrophy and death, first of the tissue elements and then of the whole body. Life being the excess of anabolism over katabolism, death is the excess of katabolism over anabolism. In discussing the question whether artificial means will ever be found for preventing a katabolic excess, or inducing an anabolic one, Allchin refers to Loeb's well-known experiments in fertilisation. In these experiments he showed that in certain ova segmentation occurred before fertilisation, and that the latter process could be dispensed with by supplying the ovum with certain inorganic salts, a perfect parthenogenesis being the result. This is attributed to ionic action, and certainly suggests that it may some day be possible for us to influence nutrition in a much more profound manner than is at present possible. A most important recent contribution to our knowledge of the physiology of nutrition has been Starling and Bayliss's discovery of secretin; an account of this substance is given in Starling's Croonian lectures.[5] Since the discovery by Ludwig of the reflex path from the mucous membrane of the mouth through the central nervous system to the secreting glands, it has been generally assumed that all the secreting processes of the digestive tract were similarly controlled. Pawlow's recent experiments gave support to the theory of central nervous reflexes, except in the case of the succus entericus, which is evoked by two processes: (1) The distension of the gut; (2) the introduction of inert pancreatic juice. Later Wertheimer and Popielski showed independently that pancreatic juice could be secreted reflexly without the intervention of the central nervous system, and they regarded it as a type of "peripheral" reflex. Starling and Bayliss, however, have succeeded in producing a secretion of pancreatic juice on the introduction of acid into the duodenum, when both the pancreas and the duodenal loop were completely isolated from each other and from the central nervous system. Subsequently Wertheimer was able to provoke a secretion of pancreatic juice in a dog by introducing acid into the duodenum of another dog, the blood of whose intestinal veins was conducted into the blood-stream of the first. Starling attributes this phenomenon to the action of a substance named secretin, which is formed as a result of the stimulation of the mucous membrane of the gut, and is carried by the blood to the pancreas, where it exercises its function of promoting secretion. Starling and Bayliss have prepared secretin from the mucous membrane of the duodenum, and though its chemical structure has not been yet determined, it is known that it is a substance resembling adrenalin, and is found to be identical in many vertebrates. It is not altered by boiling. It is not found in the mucous membrane of the duodenum unless the latter has been acted on by acids, probably a hydrolysis of a pro-secretin or inactive precursor of secretin occurs. The pro-secretin is very insoluble, is not destroyed by the death of the cell, and has not been isolated. At present, therefore, pro-secretin is a hypothetical substance. Incidentally Starling explains the intermittent closure of the pylorus by supposing that as long as the contents of the duodenum are acid the pylorus remains contracted, but when sufficient pancreatic juice has been poured out in response to the action of secretin to neutralise the chyme in the duodenum the pylorus opens and allows a further quantum of acid substances to enter the duodenum and provoke further formation of secretin. Cannon[6] tried to explain the pyloric relaxation as dependent on the presence of free HCl. in the stomach, but Einhorn[7] showed the insufficiency of this theory, as it does not explain the relaxation of the sphincter in cases such as Achylia gastrica when no HCl. is formed. Any acid, such as lactic, would, of course, be sufficient to call forth secretin from the duodenum.

**Ferments in Fruit.**—In an interesting little paper Sharp[8] details the result of his experiments on the digestive action of fruits. He finds that fresh fruits, or fruits which have not been exposed to high temperatures, or cooked for a long time possess ferments capable of digesting various proteids such as egg or serum albumin. Strawberries and pears seem to possess this power more than apples or cherries, but from the latter a dried preparation of the ferment in an impure state can be obtained. Bananas are almost inert, and should be regarded as a food, and not as an aid to digestion. Sharp concludes that ripe fresh fruit should be eaten after meals to obtain their full effects. This is in accordance with modern custom, as with that of the ancient Romans. Sharp also notes that to obtain the full laxative effect of fruits, such as stewed prunes, they should be eaten before breakfast, and not after a meal, a fact known to many mothers in the humbler ranks of society.

[3] Med. Rec., June 17, 1905, p. 952. [4] Lancet, April 29, May 6, May 13, May 20, 1905. [5] Ibid., August 5, 1905; August 12, 1905. [6] Med. Rec., June 17, 1905, p. 952. [7] Ibid. [8] Lancet, July 22, 1905, p. 219.

